# A reference set of clinically relevant adverse drug-drug interactions

**DOI:** 10.1038/s41597-022-01159-y

**Published:** 2022-03-04

**Authors:** Elpida Kontsioti, Simon Maskell, Bhaskar Dutta, Munir Pirmohamed

**Affiliations:** 1 Department of Electrical Engineering and Electronics, University of Liverpool, Liverpool, UK; 2 Patient Safety Center of Excellence, Chief Medical Office Organization, AstraZeneca Pharmaceuticals, Gaithersburg, MD USA; 3 The Wolfson Centre for Personalized Medicine, MRC Centre for Drug Safety Science, Department of Pharmacology and Therapeutics, Institute of Systems, Molecular and Integrative Biology, University of Liverpool, Liverpool, United Kingdom

**Keywords:** Outcomes research, Adverse effects, Data mining, Drug safety

## Abstract

The accurate and timely detection of adverse drug-drug interactions (DDIs) during the postmarketing phase is an important yet complex task with potentially major clinical implications. The development of data mining methodologies that scan healthcare databases for drug safety signals requires appropriate reference sets for performance evaluation. Methodologies for establishing DDI reference sets are limited in the literature, while there is no publicly available resource simultaneously focusing on clinical relevance of DDIs and individual behaviour of interacting drugs. By automatically extracting and aggregating information from multiple clinical resources, we provide a scalable approach for generating a reference set for DDIs that could support research in postmarketing safety surveillance. CRESCENDDI contains 10,286 positive and 4,544 negative controls, covering 454 drugs and 179 adverse events mapped to RxNorm and MedDRA concepts, respectively. It also includes single drug information for the included drugs (i.e., adverse drug reactions, indications, and negative drug-event associations). We demonstrate usability of the resource by scanning a spontaneous reporting system database for signals of DDIs using traditional signal detection algorithms.

## Abstract (summary)

**Table:** 

Measurement(s)	Adverse Event
Technology Type(s)	digital curation
Sample Characteristic - Organism	Homo sapiens

Machine-accessible metadata file describing the reported data: 10.6084/m9.figshare.16681933

## Background & Summary

Polypharmacy (i.e., the concomitant use of multiple medications in an individual) has become a common phenomenon in the Western world. In the United States, between 2015 and 2018, it has been estimated that two out of three people over 65 take at least three prescription medications during the course of a month (up from one third in the early 1990s), with four out of ten taking five or more medications^1^. As life expectancy is increasing around the world, leading to more people living with multiple chronic diseases, together with new medicines being launched onto the market each year, giving rise to a growing volume of possible drug combinations, the implications of drug-drug interactions (DDIs) in clinical practice have become a matter of concern. A DDI can lead to the potentiation or antagonism of one drug by another, or cause another effect that is not related to the individual drug profiles. From a mechanistic perspective, DDIs are classified into two main categories: pharmacodynamic and pharmacokinetic^2^. Predicted DDIs based on pharmacological knowledge (i.e., possible drug effect alterations caused when multiple drugs are simultaneously administered) far outnumber those with clinically significant consequences, i.e., those ranging from lack of efficacy to serious and life-threatening adverse reactions^3^. The necessarily limited time and extent of pre-clinical studies and pre-marketing clinical trials may burden the identification of adverse drug reactions (ADRs) caused by single drugs or drug combinations (i.e., adverse DDIs).

Postmarketing safety surveillance (pharmacovigilance) is a vital stage in the lifecycle management of a medicine: The number of people exposed to medicines after marketing is substantially larger than the number of volunteers involved in pre-market clinical trials. Spontaneous reporting system (SRS) databases, such as the US Food and Drug Administration (FDA) Adverse Event Reporting System (FAERS), are a particularly useful source of information, with more recent efforts focusing on the integration of multiple data sources^4–6^. Given the growing size and complexity of those databases, automated statistical tools, known as signal detection algorithms (SDAs), have become indispensable tools in an effort to distinguish real signals (i.e., information-bearing patterns) from accompanying random patterns in the background (called noise) that distract from the information^7^.

The early and effective identification of drug safety signals is of paramount importance for the pharmaceutical industry and regulatory authorities. For adverse reactions caused by DDIs, there is an increasing need for improved SDAs, with the existing state-of-the-art being less mature compared to the well-established algorithms used for detecting signals of ADRs caused by a single drug^8–10^. Even after drug approval, detection of novel DDIs might be delayed and difficult, due to the inherent complexity of DDIs, dose-dependency (i.e., some interactions only become evident in elevated drug levels) and natural human inter-variability (as well as intra-variability, in some cases) that accounts for the onset of some DDIs (e.g., rapid and poor metabolizers, genetic subpopulations, etc.). The growing volume of real-world health data presents both a challenge and an opportunity for the pharmacovigilance community, making manual review impossible and requiring a higher level of automation in the methods that are routinely used for scanning such databases.

As the available evidence is not static, the lack of gold standards (i.e., definitive positive and negative controls) poses a challenge when it comes to defining appropriate reference sets in pharmacovigilance for performance evaluation. Also, much disagreement exists in terms of the choices and criteria under consideration (e.g., well-established versus emerging cases)^11–13^.

For single drugs, a number of reference sets exist that include drug-event pairs that are either well-established (e.g., OMOP reference set^14^, Harpaz^15^), belong to recent product labelling changes^16^, or can be found in product labels (e.g., EU-ADR initiative^17^). More recently, efforts to automate the generation of a reference set for single-drug ADRs by combining multiple sources of evidence identified a number of limitations, including: size; consideration of a single data source for extracting positive controls; availability (i.e., not being open access); and inclusion of only a limited number of drugs and adverse events (AEs)^18^. Our understanding is that a fair algorithm comparison requires testing on a large reference set to derive performance metrics that are likely to indicate performance in the context of novel signals.

To the best of our knowledge, there is no established reference set for DDIs coupled with information on the individual behaviour of interacting drugs. Were such a reference set to exist, it could enable the classification of positive controls based on the possible underlying mechanism causing the interaction. Initial efforts for detecting signals indicative of DDIs included test cases that were limited in either size^9^ or variety of drugs and AEs^10^. A more advanced approach was implemented by Juhlin *et al*.^19^, although their reference set relied on a single clinical resource, which might not be a good idea since discordance among DDI compendia has been identified in the literature^20–24^.

**Fig. 1:**
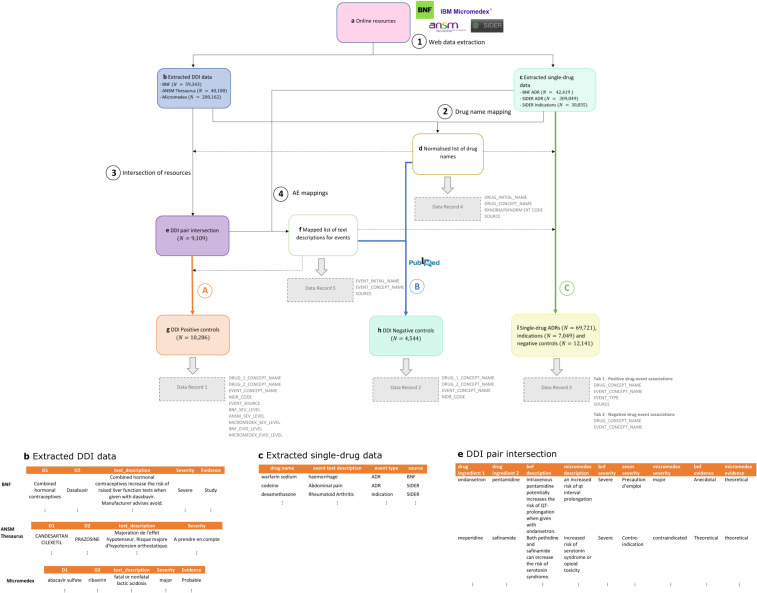
Data analysis workflow to generate positive and negative controls from DDI online resources with associated evidence for their component drugs. (1) DDI and single-drug online resources data (**a**) are extracted and stored to separate tables (**b**,**c**). (2) Drug names are normalized (**d**). (3) The intersection of the DDI online resources is extracted to a different table (**e**) and (4) English language text descriptions (for DDIs and single-drug ADRs) are annotated for AEs (after drug name masking) (**f**). (A) DDI pairs from (**e**) are assigned AEs based on the description mappings (**g**). Positive controls are published in Data Record 1. (B) Negative controls are generated using drugs and AEs from (**g**), ensuring that drug pairs cannot be found in (**b**) or in PubMed using a customized query (**h**). Negative controls are published in Data Record 2. (C) The filtered set of drugs from (**d**) is linked to AE and indication concepts using available evidence from (**c**) and negative controls for single drugs are generated following a similar process to the one described above for DDIs (i.e., drug-event is not an ADR mentioned in (**c**) or in PubMed using a customized query) (**i**). ADRs, indications and negative controls for single drugs are published in Data Record 3. Data Records 4 and 5 contain mappings of drug names and text descriptions for events, respectively. Column headers appear in grey font next to each Data Record box. Sample records from (**b**), (**c**) and (**e**) can be found in the bottom part of the figure.

Although a definitive reference standard including the complete set of DDIs cannot exist, the automatic extraction and aggregation of information from multiple clinical resources on DDIs and the individual behaviour of interacting drugs, along with scanning the scientific literature for negative controls, enabled us to construct, share, and advocate CRESCENDDI (**C**linically-relevant **RE**ference **S**et **CEN**tered around **D**rug-**D**rug **I**nteractions), a dataset that can be used to facilitate research in SDAs and allow common ground for comparing methodologies. We propose a scalable approach for generating a normalized reference set that requires less manual effort for future updates, considering the dynamic nature of data and evidence availability, and for subset selection using design criteria. Figure 1 outlines the main steps for generating the reference set.

## Methods

The processing pipeline for the construction of CRESCENDDI included the following steps: (1) web data extraction of information related to DDIs and single-drug ADRs from 4 different online resources for DDIs, single-drug ADRs, and drug indications; (2) mapping (normalization) of drug names appearing in the extracted data; (3) extraction of the intersection of the DDI online resources; (4) manual annotation and mapping of English language text descriptions for DDIs, single-drug ADRs, and drug indications to MedDRA concepts; (5) generation of positive controls for DDIs using the normalized intersection of the DDI online resources; (6) generation of negative controls using drugs and AEs from the positive control set combined with PubMed search; and (7) aggregation of information on single-drug ADRs and drug indications for the drugs appearing in the DDI reference set (i.e., positive and negative control sets) and generation of negative controls for single drugs with a process similar to the one followed in the previous step for DDI negative controls.

### Web data extraction

DDI data were derived from the following online resources: the British National Formulary (*BNF*) website^25^, the French National Drug Safety Institute (ANSM) Portable Document Format (PDF) file (*Thesaurus*)^26^ and the *Micromedex* platform^27^. For *BNF* and *Micromedex*, web data extraction tools in Python 3.6^28^ enabled the extraction of the relevant fields into a Comma Separated Values (CSV) file (June-August 2018). For *Thesaurus*, the R package IMThesaurusANSM^29^ was used and the resulting R dataframe from the 2019 update was converted to a CSV file.

The tables contained the following fields:

- interacting drug name 1 (*D1*) (e.g., *Metoprolol Tartrate*);
- interacting drug name 2 (*D2*) (e.g., *Lidocaine*);
- text description for the DDI (e.g., *Lidocaine is predicted to increase the risk of cardiovascular adverse effects when given with metoprolol. Manufacturer advises use with caution or avoid*);
- severity label (e.g., *Severe*);
- evidence label (if available) (e.g., *Study*).

For single-drug ADRs, the following sources were considered: the BNF website^25^ and SIDER dataset^30^. For drug indications, SIDER was used. BNF ADR data were extracted in a similar way as previously with DDI data (automated web data extraction) into a CSV file, while SIDER data for ADRs and drug indications were already available in CSV files.

A table containing the following fields was constructed:

- drug name (e.g., *Metoprolol Tartrate*);
- event text description (e.g., *Bradycardia*);
- event type (e.g., *ADR*);
- source (e.g., *SIDER*).

### Drug name mapping

To facilitate usability and ensure compatibility, a standardization process was followed such that we could provide a resource with normalized concepts to standard terminologies for drugs and medical events. Specifically, the Observational Health Data Sciences and Informatics (OHDSI) Vocabulary version 5 was selected for mapping the drug names occurring in each of the DDI online resources into RxNorm and RxNorm Extension standard codes (at the Ingredient level) using OHDSI Usagi^31^.

We removed combination drugs (as DDIs of their constituent drug ingredients were separately mentioned), vaccines, vitamins, herbal medicines, food, beverages, supplements, tobacco, and lab tests. Also, generic drug classes (e.g., combined hormonal contraceptives, hormonal replacement therapy) appearing in the BNF were not mapped to their individual drug ingredients, as there was no table on the BNF website specifying the drugs belonging in each drug class. We mapped the remaining unique drug names occurring in the DDI resources to OHDSI standard vocabulary concept identifiers. For example, *Metoprolol Tartrate* was mapped to the RxNorm Ingredient concept *metoprolol*. For Thesaurus, a native French speaker (pharmacist) confirmed the drug mappings of French drug names to English language OHDSI concepts.

A similar process was followed for drugs that appear in the single-drug data (ADRs and indications).

### Intersection of DDI online resources

By matching drug names to their mapped drug ingredients in the extracted DDI data tables, we obtained the set of common drug pairs across the tables and generated a new table that contains only the DDIs and associated information which could be found in each of the DDI online resources under consideration. Cases where the interacting drug mapping of *D*1 and *D*2 were swapped in the original data tables (i.e., (*D*1,*D*2) and (*D*2,*D*1)) were considered equivalent.

The final table contained the following fields:

- drug_1 concept name (e.g., *metoprolol*);
- drug_2 concept name (e.g., *lidocaine*);
- bnf description (e.g., *Lidocaine is predicted to increase the risk of cardiovascular adverse effects when given with metoprolol. Manufacturer advises use with caution or avoid*);
- micromedex description (e.g., *lidocaine toxicity (anxiety, myocardial depression, cardiac arrest)*);
- bnf severity (e.g., *Severe*);
- ansm severity (e.g., *Précautions d’emploi (Precautions for use)*);
- micromedex severity (e.g., *major*);
- bnf evidence (e.g., *Study*);
- micromedex evidence (e.g., *probable*).

### Adverse event and indication mappings

For DDI-related text descriptions in English from BNF and Micromedex that could be found in the DDI intersection table, a drug name blinding process was performed by replacing the interacting drug names with a common token in all cases (i.e., ‘X’). In this way, the number of unique descriptions was reduced, thus facilitating the mapping process that followed. For example, the descriptions:

*Both dexibuprofen and ibuprofen can increase the risk of nephrotoxicity*.and

*Both polymyxins and streptomycin can increase the risk of nephrotoxicity*.

were both mapped to the following blinded description:

*Both X and X can increase the risk of nephrotoxicity*.

The set of blinded text descriptions for BNF and Micromedex was extracted from the table and a semi-automated mapping process using OHDSI Usagi mapped them to MedDRA PT concepts. We explicitly focused on text descriptions that included clinical manifestations of DDIs, e.g., *X may increase the risk of hypoglycaemia when taken with X*. Text descriptions containing a potential mechanism of the interaction were left unmapped. In some cases, a single text description was linked to multiple concepts. For example:

*Interaction Effect*: *An increased risk of cardiotoxicity (QT prolongation*, *torsades de pointes*, *cardiac arrest)*.

includes 3 different MedDRA PTs.

Also, serotonin syndrome was not mapped to its corresponding MedDRA PT and was not further considered as an AE for inclusion in the reference set.

Text descriptions from the BNF regarding single-drug ADRs were mapped to MedDRA PTs (where possible), but only for the drug ingredients that could be found in the DDI pair intersection table. SIDER ADR and indication data for the same list of drugs were also mapped to OHDSI concepts; however, for this resource, MedDRA PT codes were already available.

### Positive controls

The set of positive controls was derived from the DDI intersection table, using mappings of text descriptions to AEs that were generated in the previous step. It contained 10,286 drug-drug-event (DDE) triplets, 454 unique individual drug ingredients, and 179 unique AEs (as OHDSI concepts) in total.

### Negative controls

The set of negative controls was generated by randomly pairing two drug ingredients from the 454 unique drug ingredients that can be found in the positive controls and, in case the random drug pair did not appear in any of the DDI online resources, then it was randomly paired with an AE from the 179 unique AEs present in the positive control set. The choice of generating negative controls with common drug ingredients and AEs as the ones appearing in positive controls aimed to ensure the generation of a balanced reference set that does not contain added biases by design. For each of the created DDE triplets, a customized query (“(**DRUG_1_CONCEPT_NAME**) AND (**DRUG_2_CONCEPT_NAME**) AND ((**EVENT_CONCEPT_NAME**) OR (interaction))”, e.g., “(Oxazepam) AND (Naproxen) AND ((Hyperkalaemia) OR (interaction))”) was submitted to PubMed in an automated fashion and, if the search returned no results, the triplet was added to the negative control set. This process aimed to provide more confidence, to the best of our ability, about the absence of literature evidence of a potential DDI for the triplet under consideration, rather than definitive evidence to support the lack of a potential association.

The process was repeated until the number of negative controls with non-zero counts in the US Food and Drug Administration (FDA) Adverse Event Reporting System (FAERS) database (see Usage Notes) was similar in size (N = 4,544) compared to the equivalent subset of positive controls. The negative control set included 161 unique AEs and 435 unique drug ingredients.

### Single-drug ADRs, indications, and negative controls

By replacing text descriptions from BNF (N = 1,538) and MedDRA PT codes to their corresponding mapped OHDSI concepts, a table with ADR and indication information related to the drug ingredients of the DDI reference set was generated. The table included: 438 unique drug ingredients, which could be found in at least one of the resources under consideration (i.e., BNF and SIDER), 3,492 AEs and 1,557 indication terms (as OHDSI concepts).

BNF and SIDER jointly contained 69,721 single-drug ADRs, with 12,318 common instances; this set could be utilized as a source for single-drug positive controls. This set covered 381 unique drugs and 835 unique AE concepts. Random pairing of those drugs and AE concepts followed by submission of a customized query (“(**DRUG_CONCEPT_NAME**) AND (**EVENT_CONCEPT_NAME**) AND ((adverse event) OR (adverse drug reaction))’”, e.g., “(oxazepam) AND (myoclonus) AND ((adverse event) OR (adverse drug reaction))”) to PubMed (to ensure absence of literature evidence of a potential ADR for the various drug-event associations) enabled the generation of a negative control set for single drugs (N = 12,141).

## Data Records

CRESCENDDI is publicly available online through Figshare^32^ in 5 Excel spreadsheets. The fields contained in each of the spreadsheets are outlined below.

### Data Record 1: Positive controls

DRUG_1_CONCEPT_NAME: Name of the first drug (active ingredient) that comprises a test case (DDE triplet).

DRUG_2_CONCEPT_NAME: Name of the second drug (active ingredient) that comprises a test case (DDE triplet).

EVENT_CONCEPT_NAME: Name of the normalized event (as a MedDRA PT) that comprises a test case (DDE triplet).

MDR_CODE: MedDRA code associated with the PT specified in the EVENT_CONCEPT_NAME field.

EVENT_SOURCE: Label indicating the resource where the event is mentioned (i.e., ‘BNF’, ‘Micromedex’, ‘BNF + Micromedex’).

BNF_SEV_LEVEL: Label with the values of ‘Severe’, ‘Moderate’ or ‘Mild’ that indicates the severity level associated with the DDI as shown in BNF (if available).

ANSM_SEV_LEVEL: Label with the values of ‘Contraindicated’, ‘Not recommended’, ‘Precautions for use’ or ‘Take into consideration’ that indicates the severity level associated with the DDI as shown in Thesaurus.

MICROMEDEX_SEV_LEVEL: Label with the values of ‘Contraindicated’, ‘Major’, ‘Moderate’ or ‘Minor’ that indicates the severity level associated with the DDI as shown in Micromedex.

BNF_EVID_LEVEL: Label with the values of ‘Study’, ‘Anecdotal’ or ‘Theoretical’ that indicates the evidence level associated with the DDI as shown in BNF (if available).

MICROMEDEX_EVID_LEVEL: Label with the values of ‘Established, ‘Theoretical’ or ‘Probable’ that indicates the evidence level associated with the DDI as shown in Micromedex.

### Data Record 2: Negative controls

DRUG_1_CONCEPT_NAME: Name of the first drug (active ingredient) that comprises a test case (DDE triplet).

DRUG_2_CONCEPT_NAME: Name of the second drug (active ingredient) that comprises a test case (DDE triplet).

EVENT_CONCEPT_NAME: Name of the normalized event (as a MedDRA PT) that comprises a test case (DDE triplet).

MDR_CODE: MedDRA code associated with the PT specified in the EVENT_CONCEPT_NAME field.

### Data Record 3: Single-drug ADRs, indications and negative controls

**Tab 1 – Positive drug-event associations.**

**Table 1:** Statistics related to the performance evaluation of three SDAs for DDIs using FAERS data.

Citation	SDA	Performance analysis in the original source	AUC (95% CI)	Medians	
				Score+	Score−
*Noren et al., 2008* ^8^	Omega	a. 5 positive examples (case studies); b. World Health Organization (WHO) database-wide screen	0.5670 (0.5534, 0.5806)	−1.6173	−2.3151
*Thakrar et al., 2007* ^9^	delta_add	4 positive and 4 negative controls tested on FAERS	0.4211 (0.4110, 0.4312)	0.00078	0.00417
*Almenoff et al., 2003* ^10^	IntSS	Beta blockers + Verapamil (as positive controls) and Angiotensin-converting enzyme (ACE) inhibitors/Angiotensin-2 receptor blockers + Verapamil (as negative controls) tested on FAERS for impaired myocardial conduction	0.5041 (0.4921, 0.5162)	0.3669	0.3453

AUC, area under the receiver operating characteristic curve; Score+, median signal score for the set of positive controls; Score−, median signal score for the set of negative controls.

DRUG_CONCEPT_NAME: Name of the drug (active ingredient) that comprises a single drug information case (drug-event pair).

EVENT_CONCEPT_NAME: Name of the normalized event (as a MedDRA PT) that comprises a single drug information case (drug-event pair).

EVENT_TYPE: Type of association between the drug and event of the single drug information case (i.e., ‘Adverse event’ or’Indication’).

SOURCE: Label indicating the resource of the single drug information case (i.e., ‘BNF’ or ‘SIDER’).

**Tab 2 – Negative drug-event associations.**

**Table 2:** Statistics related to the performance evaluation of three SDAs for single drugs using FAERS data.

Citation	SDA	Resource union Positive controls: 52,637 Negative controls: 12,141	Resource intersection Positive controls: 12,318 Negative controls: 12,141
		AUC (95% CI)	AUC (95% CI)
*Evans et al., 2001* ^34^	Proportional Reporting Ratio (PRR)	0.5791 (0.5746, 0.5835)	0.5959 (0.5884, 0.6033)
*DuMouchel, 1999* ^35^	EBGM (Empirical Bayes Geometric Mean)	0.6308 (0.6259, 0.6356)	0.6593 (0.6510, 0.6675)
*Bate et al., 1998* ^36^	BCPNN (Bayesian Confidence Propagation Neural Network)	0. 7063 (0.7008, 0.7117)	0.7495 (0.7401, 0.7588)

AUC, area under the receiver operating characteristic curve; Score+, median signal score for the set of positive controls; Score−, median signal score for the set of negative controls.

DRUG_CONCEPT_NAME: Name of the drug (active ingredient) that comprises a single drug negative case (drug-event pair).

EVENT_CONCEPT_NAME: Name of the normalized event (as a MedDRA PT) that comprises a single drug negative case (drug-event pair).

### Data Record 4: Drug mappings

DRUG_INITIAL_NAME: Unmapped name of the drug as extracted from the resource.

DRUG_CONCEPT_NAME: Normalized name of the drug (as an RxNorm Ingredient) extracted from the resource.

RXNORM_CODE/RXNORM_EXTENSION_CODE (OHDSI): RxNorm/RxNorm Extension code associated with the normalized drug name specified in the DRUG_CONCEPT_NAME field.

SOURCE: Label indicating the resource where the drug is found (i.e., ‘BNF_DDI’, ‘Thesaurus_DDI’, ‘Micromedex_DDI’, ‘BNF_Single’).

### Data Record 5: Event mappings

EVENT_INITIAL_TEXT: Text description (after drug name blinding) containing an AE as extracted from the resource.

EVENT_CONCEPT_NAME: Mapped name of the event (as a MedDRA term) extracted from the resource.

SOURCE: Label indicating the resource where the text description is found (i.e., ‘BNF_DDI’, ‘Micromedex_DDI’, ‘BNF_Single’).

## Technical Validation

Given the inability to generate ‘gold standards’ in pharmacovigilance, the validation of the reference set included steps that supported the technical quality of the procedures followed to generate the dataset, rather than attempting to further ensure the validity of each control. The process of validating the reference set was two-way.

First, we verified the original online data as well as the unmapped extracted DDI data versus the curated reference set in order to validate the accuracy of the automated extraction and concept mapping processes. A random sample of 40 positive and 40 negative controls was manually checked in each of the DDI online resources (i.e., *BNF*, *Thesaurus﻿,* and *Micromedex*), to ensure the presence or absence, respectively, of the DDE triplet in the information provided. Due to the time lag between data retrieval (June-August 2018) and validation (September 2021), there were issues most probably related to differing versions of the resources. More specifically, there were 6 positive controls missing from one of the resources (5 from the BNF and 1 from Micromedex), 2 of which due to the complete removal of the drug monograph. Also, one of the negative control samples had been added to the BNF. No issues were identified during the validation of the random sample of controls from the curated reference set against the unmapped extracted DDI data.

Second, the validation of text description mapping for AEs included independent annotation of a random sample of 100 text descriptions for AEs to ensure agreement on event mapping. No issues were identified.

Expert manual review of a number of cases from the reference set could be potentially performed as an additional layer of the technical validation procedure to confirm that the included DDIs are worthy of note. We have attempted to replace this need for expert manual review by combining information from multiple resources of clinical interest.

The next section outlines the application of the reference set for drug safety surveillance in an SRS database using SDAs that have been described in previous studies, in an effort to showcase its validity through practical implementation as well as prove its potential use in the real world.

## Usage Notes

This publicly available resource aims to provide a common ground for evaluation of SDAs related to DDI signals, with a focus on assembling information from disparate sources that could provide support toward the clinical relevance of controls. The size of the reference set alongside the relatively large number of drugs and AEs considered enables a quantitative approach in algorithm performance evaluation in terms of SDAs developed for DDI signals.

Also, the supplementary single-drug positive and negative control sets could be used separately for signal detection in the context of adverse drug-event associations.

This reference set could be applied to a variety of data sources (e.g., electronic health record data, social media, literature). Here, we illustrate applicability in one such context.

### FAERS screening for DDI signals

In pharmacovigilance, signal detection is largely based on methodologies that use disproportionality analysis, meaning that the observed counts are compared to the expected ones, assuming that the drug(s) and the events occur independently. For DDIs, similar measures of disproportionality have been described in the literature. For our analysis, the following statistical measures were considered:

(i) An observed-to-expected shrunk interaction measure (*Omega*)^8^
(ii) The ‘interaction coefficient’ in a linear regression model with additive baseline (*delta_add*)^9^
(iii) A measure based on an adapted version of Multi-Gamma Poisson Shrinker (MGPS) model, called Interaction Signal Score (*IntSS*)^10^

A curated and standardized version of FAERS was used as the test data source. In FAERS, each spontaneous report contains information on administered drugs, AEs experienced, indications, and demographic information (i.e., sex, age, etc). Using a slight modification of the Adverse Event Open Learning through Universal Standardization (AEOLUS) process that was described in published work^33^, FAERS data files corresponding to the period 2004Q1–2018Q4 were standardized to the RxNorm Ingredient level terms and MedDRA PTs, so that compatibility of the reference set with the test data could be established. The total number of reports containing at least one drug and one AE was 9,203,239.

ROC analysis was performed using MATLAB function perfcurve. 95% confidence interval (CI) estimates for the AUC were calculated using leave-one-out cross validation.

Table 1 shows statistics related to the performance of the SDAs in FAERS using our advocated reference standard. For each algorithm, the table provides the AUC scores with 95% CI estimates. As opposed to expectations, only two out of three SDAs for DDIs (i.e., Omega and IntSS) performed better than random guessing. Median signal scores for the set of positive controls were larger compared to those of negative controls in those two cases, which provides support for the validity of CRESCENDDI. Limited performance analyses in the studies describing the SDAs for DDIs as well as absence of other efforts in the literature for quantitative performance assessment across multiple thresholds did not manage to provide an appropriate benchmark for this study.

### FAERS screening for single-drug ADRs

For the single-drug reference set, empirical support was provided by applying three well-established SDAs to FAERS data. Positive controls (at the MedDRA PT level) were sourced from either the intersection (N = 12,318) or the union (N = 52,637) of the resources under consideration (i.e., BNF and SIDER) and ROC analysis for performance evaluation similarly to the previous section (Table 2).

Table 2 shows that *EBGM* outperformed *PRR*, which is in line with the results of other studies^14^. Also, improved performance was noticed in the case of resource intersection for all SDAs, which advocates the use of information that appears in multiple resources rather than relying on a single data source for deriving positive controls.

### Flexibility of the resource

This reference set has been mapped to standard vocabularies using the OHDSI framework, thus enabling linking to other ontologies and/or vocabularies, based on research needs, test data resources format, etc.

Considering the changing nature of available evidence for both positive and negative controls, reproduction of the resource in the future would be recommended to ensure that it is up to date. Although specific products (e.g., vaccines) were excluded given our focus on generating a reference set relevant to DDIs, slight modifications on the drug mapping process can allow the inclusion of controls pertinent to any additional products of interest.

The incorporation of additional information (e.g., shared indication, single-drug AE under the same HLT/HLGT MedDRA level, evidence levels) enables filtering and stratification of controls. The effect of stratification using those design criteria is outside the scope of this paper, but we aim to explore this aspect in future research.

## Data Availability

The code used to generate this dataset is publicly available on a GitHub repository (https://github.com/elpidakon/CRESCENDDI). This code was developed and tested using: OHDSI standard vocabulary version v5.0 18-JAN-19 (https://athena.ohdsi.org/), which includes: RxNorm version 20181203, RxNorm Extension version 2019-01-17, and MedDRA version19.1. Database storage and operations were enabled using PostgreSQL 9.3. Drug and event mapping steps were performed using OHDSI Usagi version 1.2.7 (https://github.com/OHDSI/usagi). Web data extraction was performed using Python 3.6. Scores for the SDAs were calculated using Python 3.6 (Omega), SAS (delta_add) and R version 4.0.0 (IntSS, PRR, EBGM and BCPNN); AUC scores and CI estimates were calculated using MATLAB R2020b (perfcurve function).
